# Changes in the intestine microbial, digestion and immunity of *Litopenaeus vannamei* in response to dietary resistant starch

**DOI:** 10.1038/s41598-019-42939-8

**Published:** 2019-04-23

**Authors:** Yafei Duan, Yun Wang, Qingsong Liu, Hongbiao Dong, Hua Li, Dalin Xiong, Jiasong Zhang

**Affiliations:** Key Laboratory of South China Sea Fishery Resources Exploitation & Utilization, Ministry of Agriculture and Rural Affairs; Key Laboratory of Fishery Ecology and Environment, Guangdong Province, South China Sea Fisheries Research Institute, Chinese Academy of Fishery Sciences, Guangzhou, 510300 P.R. China

**Keywords:** Mucosal immunology, Microbial communities

## Abstract

Resistant starch (RS) is a constituent of dietary fibre that has beneficial effects on the intestine physiological function of animals. However, the roles of RS on shrimp intestine health is unknown. In this study, we investigated the the effects of dietary RS on the microbial composition, and digestive and immune-related indices in the intestine of *Litopenaeus vannamei*. The shrimp were fed with diets containing different levels of RS: 0 g/kg (Control), 10 g/kg (RS1), 30 g/kg (RS2) and 50 g/kg (RS3) for 56 days. The results showed that dietary RS improved the morphology of the intestine mucosa. RS also increased the activity of digestive enzymes (AMS, LPS, Tryp, and Pep) and immune enzymes (PO, T-AOC, T-NOS, and NO), and the expression levels of immune-related genes (*proPO*, *ALF*, *Lys*, *HSP70*, *Trx*, *Muc-1*, *Muc-2*, *Muc-5AC*, *Muc-5B*, and *Muc-19*). A microbiome analysis indicated that dietary RS increased the short-chain fatty acids (SCFAs) contents and altered the composition of the intestine microbial. Specifically, RS increased the abundances of Proteobacteria and decreased the abundance of Bacteroidetes. At the genus level, the beneficial bacteria (*Lutimonas*, *Ruegeria*, *Shimia*, *Mesoflavibacter*, and *Mameliella*) were enriched, which might be involved in degrading toxins and producing beneficial metabolites; while potential pathogens (*Formosa* and *Pseudoalteromonas*) were decreased in response to dietary RS. Our results revealed that dietary RS could improve the intestine health of *L*. *vannamei*, probably via modulating the intestine microbial composition and SCFAs contents, and enhancing the digestion and immunity of the shrimp.

## Introduction

The intestine microbial has a mutual relationship with its host which influences the digestibility, metabolism, and immunity of the host^1,2^. Healthy intestine microbial can protect the host from colonization by pathogenic microbes and produce short-chain fatty acids (SCFAs). Conversely, unbalanced intestine microbial (dysbiosis) may lead to the alteration of immunity, and increase the susceptibility to diseases^3,4^. SCFAs, including acetate, propionate and butyrate, are shown to improve disease resistance^5^. SCFAs can provide nutrition for intestine mucosa, and benefit the healthy intestine micro-ecological environment of the host^6^. The production of SCFAs occurs mainly through the bacterial fermentation of fibre that non-digestible by the gastrointestinal systems of animals^7^. Therefore, dietary interventions, especially via supplementation with fiber, are efficient for regulation of the intestinal microbial, will be beneficial to the host health.

Different kinds of dietary fibres have unique metabolic effects and produce different kinds and amounts of SCFAs^8^. As a constituent of dietary fibre, resistant starch (RS) is non-digestible in the intestine of animals but can be fermented by resident microbial^9^. Diets that are rich in RS have beneficial effects to animal health, especially produce SCFAs by microbial fermentation, which can acidify the intestine environment, modify and stabilize intestine microbial^10^. Other health benefits associated with dietary RS are the promotion of the growth and proliferation of intestine epithelial cells, and the induction the transcription of genes which are conducive to intestine development^11^. There are four different types of RS: (1) RS1 is a physically inaccessible starch exist in legumes and grains; (2) RS2 is a raw starch granule, including raw potatoes and green (unripe) bananas; (3) RS3 is a retrograded starch, including potatoes and rice; (4) RS4 is a chemically modified starch, including esters, ethers, and cross-linked starches. Several studies have explored the effects of RS on the intestine health of swine and poultry^12,13^. However, the roles of RS in shrimp intestine health remain unknown.

The Pacific white shrimp *Litopenaeus vannamei* is a commercially important marine species in the world. Shrimp farming has suffered from serious problems caused by the various diseases^14^. High applications of antibiotics and antimicrobial drugs easily pollute the environment, thereby resulting in bacterial resistance; thus, ecofriendly disease preventative approaches must be developed for antibiotics alternative^15^. RS is rendered as a good alternative to antibiotic. Intestine mucosa is an important barrier for shrimp disease defence, and intestine health clearly affects shrimp health^16^. Therefore, in this study, we explored the effects of dietary RS on the intestine health of *L*. *vannamei*, in terms of intestine histological structure, the digestive and immune indices, and the intestine microbial composition and metabolite SCFAs contents.

## Results

### Intestine epithelium morphology

**Figure 1:**
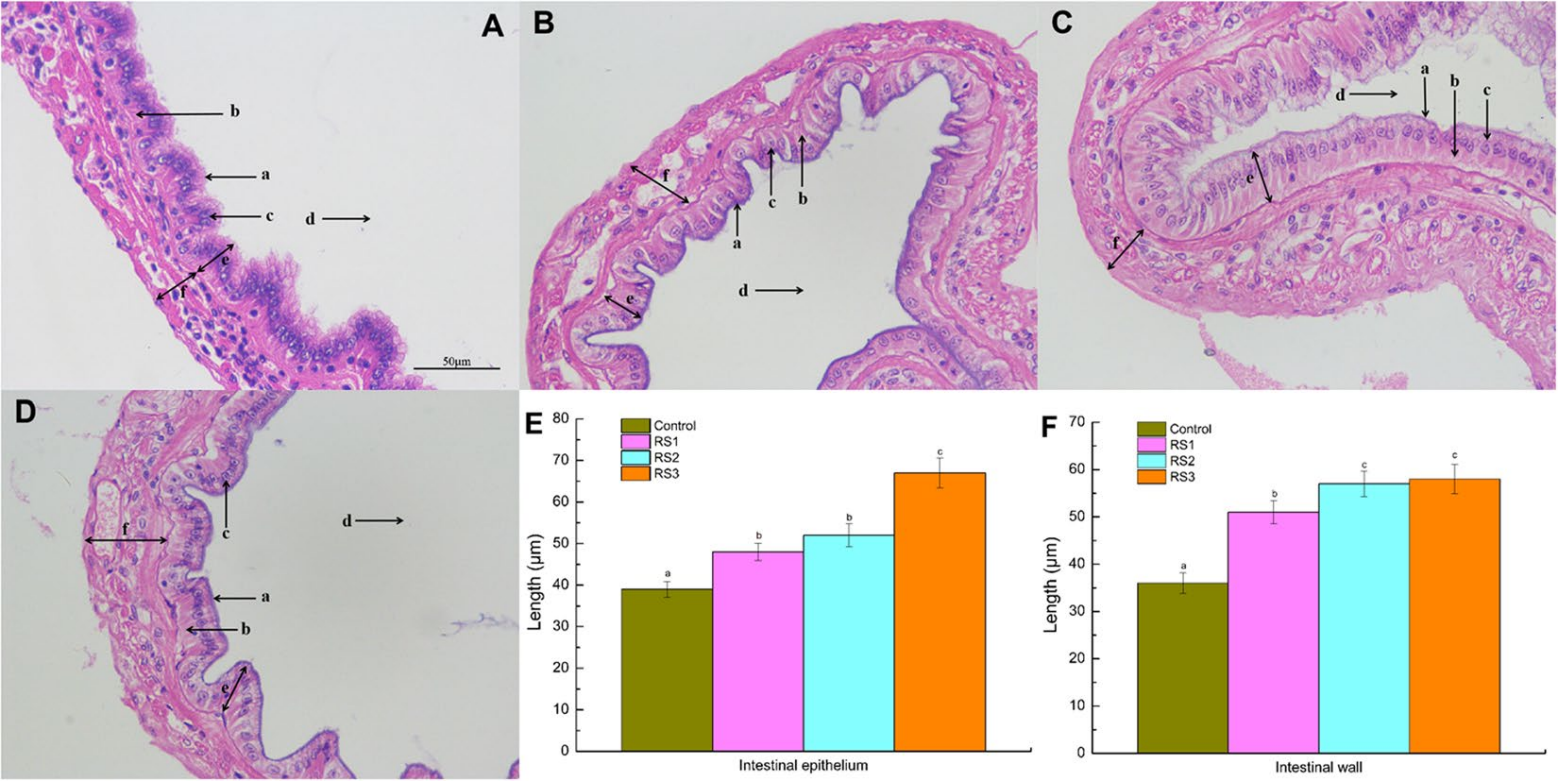
Intestine tissue of *L*. *vannamei* stained with HE after the shrimp were fed the control and three RS diets for 56 days. (**A**) Control group, × 400; (**B**) RS1 group, × 400; (**C**) RS2 group, × 400; (**D**) RS3 group, × 400. (**a**) mucosa brush border, (b) intestine epithelium, (c) cell nuclei, (d) intestine lumen, (e) epithelium height (EH), (f) wall thickness (WT). Bars show the mean ± SE (*n* = 5). The different letters (a–c) indicate significant differences (*P* < 0.05) among the groups.

Shrimp fed the RS diet had a better intestine health morphology than those of the control group (Fig. 1). Health characteristics included good epithelial cell morphology, cell density in neat rows, and higher epithelium height (EH) and wall thickness (WT) of the three RS groups than those of the control group (*P* < 0.05).

### Digestive and immune enzymes activity

**Figure 2:**
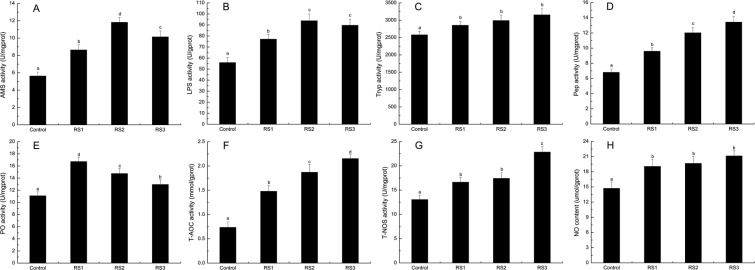
Digestive and immune enzyme indices in the intestine of *L*. *vannamei* that were fed the control and three RS diets for 56 days. (**A**) AMS; (**B**) LPS; (**C**) Tryp; (**D**) Pep; (**E**) PO; (**F**) T-AOC; (**G**) T-NOS; (**H**) NO. Bars show the mean ± SE (*n* = 3). The different letters (a–c) indicate significant differences (*P* < 0.05) among the groups.

Shrimp fed the RS diet had greater digestive and immune enzymes activity than those of the control group (Fig. 2). Amylase (AMS) activity in the RS2 group was highest (*P* < 0.05). Lipase (Lip) activity in the RS2 and RS3 groups was highest, and there were no differences between them (*P* > 0.05). The trypsin (Tryp) activity showed no differences in the three RS groups (*P* > 0.05). The pepsin (Pep) activity was increased in the three RS groups with the increasing doses of RS (*P* < 0.05). The phenoloxidase (PO) activity was increased in the three RS groups (*P* < 0.05) with the decreasing doses of RS. The total antioxidant capacity (T-AOC) activity was increased in the three RS groups (*P* < 0.05) with the increasing doses of RS. The total nitric oxide synthase (T-NOS) activity was highest in the RS3 group (*P* < 0.05), and there were no differences between the RS1 and RS2 groups. The nitric oxide (NO) content in the three RS groups was statistically the same (*P* > 0.05).

### Immune-related genes expression

**Figure 3:**
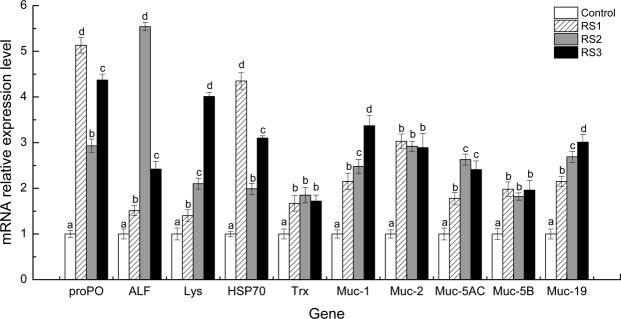
Immune genes expression in the intestine of *L*. *vannamei* that were fed the control and three RS diets for 56 days. Bars show the mean ± SE (*n* = 3). The different letters (a–c) indicate significant differences (*P* < 0.05) among the groups.

Shrimp fed the RS diet had a higher immune-related genes expression level than those of the control group, including the antibacterial, antioxidant, and mucus functions (Fig. 3). Antibacterial genes, such as prophenoloxidase (*proPO*), anti-lipopolysaccharide factor (*ALF*), and lysozyme (*Lys*), were the highest in the RS1, RS2, and RS3 groups respectively (*P* < 0.05). Antioxidant genes, such as the heat shock protein 70 (*HSP70*) was the highest in the RS1 group (*P* < 0.05), thioredoxin (*Trx*) in the three RS groups was statistically the same (*P* > 0.05). Mucin (*Muc*) genes, such as *Muc-1* and *Muc-19*, were the highest in the RS3 group (*P* < 0.05), *Muc-2* and *Muc-5B* were no differences in the three RS groups (*P* > 0.05), *Muc-5AC* was the highest in the RS2 and RS3 groups (*P* < 0.05).

### Intestine microbial richness and diversity

**Figure 4:**
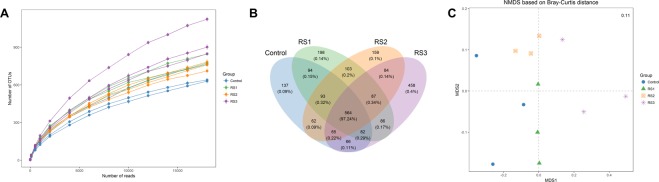
Rarefaction curves, venn diagram, and NMDS comparative of the intestine microbial of *L*. *vannamei* that were fed the control and three RS diets for 56 days. (**A**) Rarefaction curves; (**B**) Venn diagram; (**C**) NMDS based on Bray-Curtis distance.

Illumina sequencing of the intestine microbial produced 1,587,040 clean reads, and the average sequence length was 316 bp. A rarefaction curve analysis of the observed species per sample was sufficient (Fig. 4A). A total of 564 (97.24%) OTUs were co-owned by all of the groups, and the unique OTUs were higher in the three RS groups compared with those of the control group (Fig. 4B). The largest category of the unique OTUs was the RS3 group (458, 0.4%), the second was the RS1 group (198, 0.4%), and the third was the RS2 group (159, 0.1%). Non-metric multi-dimensional scaling (NMDS) based on the Bray-Curtis distance was performed to detect the relationships among the microbial in different samples and confirmed that the intestine microbial of the four groups were separated (Fig. 4C).

Similar to the OTUs number, the Chao 1, ACE, Simpson and Shannon indices of the three RS groups were all higher than those of the control group (Table 1). The Chao 1 and ACE indices of the RS1 and RS3 groups were higher than those of the RS2 group (*P* < 0.05). The Shannon index was no difference among the three RS groups (*P* > 0.05). The Simpson indices of the RS1 and RS2 groups were higher than those of the RS3 group (*P* < 0.05).

**Table 1:** Intestine microbial diversity of *L*. *vannamei* that were fed the control and three RS diets for 56 days.

Group	Observed	Chao1	ACE	Shannon	Simpson
Control	625 ± 33^a^	927.03 ± 49.34^a^	985.31 ± 18.44^a^	2.60 ± 0.17^a^	0.67 ± 0.04^a^
RS1	775 ± 14^b^	1266.70 ± 68.05^c^	1317.38 ± 21.40^c^	3.72 ± 0.16^b^	0.91 ± 0.02^c^
RS2	741 ± 20^b^	1127.10 ± 61.16^b^	1220.34 ± 20.56^b^	3.53 ± 0.06^b^	0.87 ± 0.02^c^
RS3	899 ± 28^c^	1266.75 ± 55.75^c^	1393.17 ± 21.66^c^	3.39 ± 0.04^b^	0.82 ± 0.01^b^

ACE: abundance-based coverage estimator. Values represent the mean ± SE (*n* = 3). The different letters (a–c) indicate significant differences (*P* < 0.05) among the groups.

### Changes in the intestine bacterial composition

**Figure 5:**
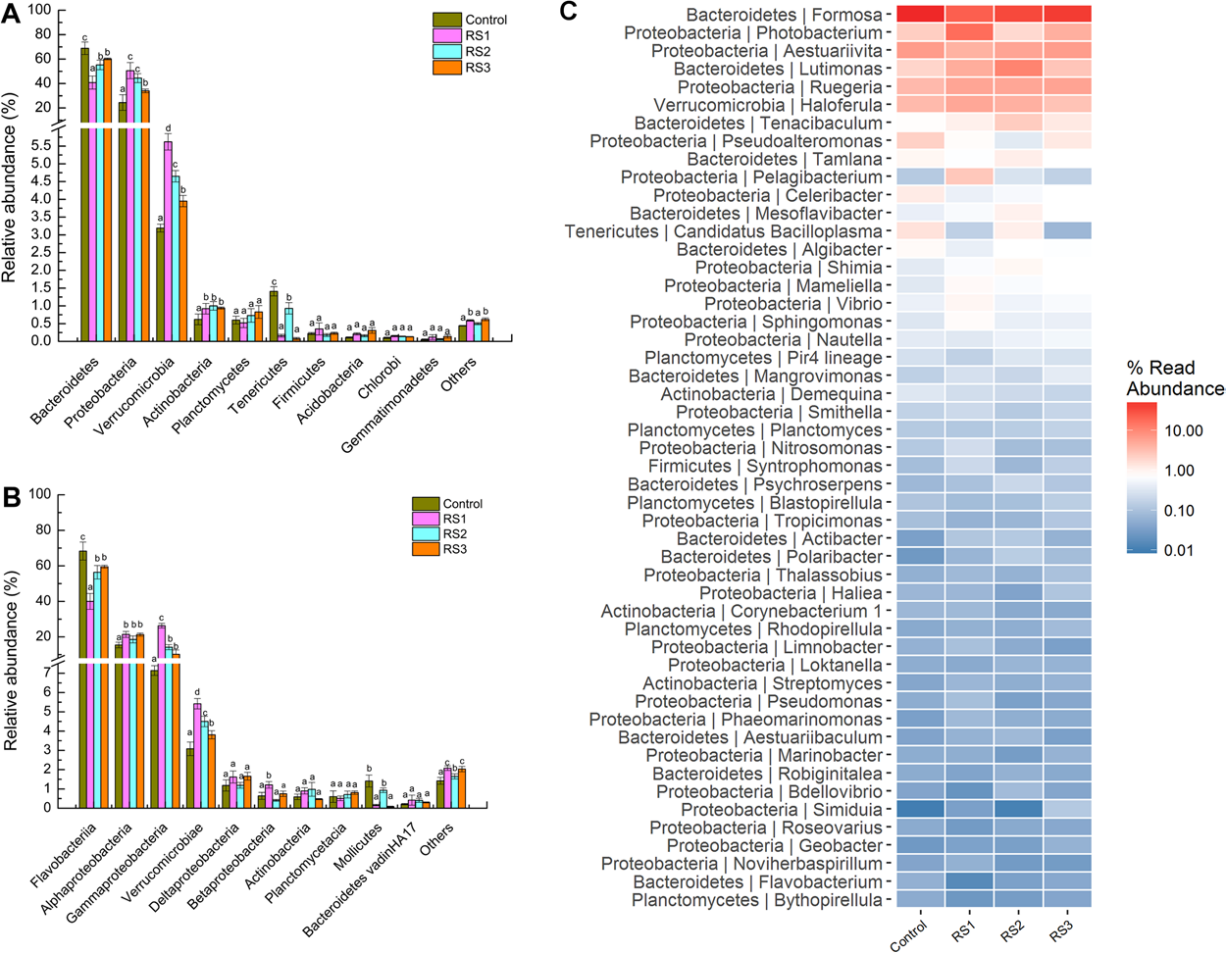
Intestine microbe composition of *L*. *vannamei* that were fed the control and RS diets for 56 days. (**A**) phylum level; (**B**) class level. Bars show the mean ± SE (*n* = 3). The different letters (a–c) indicate significant differences (*P* < 0.05) among groups. (**C**) Heatmap analysis of intestine microbial on the top 50 genera. The redder colour show the higher abundance of the genera, and the black colour is the lower abundance.

In total, 35 different bacterial phylum were identified; Proteobacteria, Bacteroidetes, and Verrucomicrobia were three of the dominant phylum. Compared with the control group, Proteobacteria, Verrucomicrobia, and Acidobacteria abundance were increased in the three RS groups, while that of Bacteroidetes and Tenericutes were decreased (Fig. 5A). At the class level, the abundances of Alphaproteobacteria, Gammaproteobacteria, and Verrucomicrobiae were increased in the three RS groups, while that of Flavobacteriia was decreased (Fig. 5B). At the genus level, the abundances of certain beneficial bacteria, *Lutimonas*, *Ruegeria*, *Shimia*, *Mesoflavibacter*, and *Mameliella*, were increased in the three RS groups, while that of some potential pathogens, such as *Formosa* and *Pseudoalteromonas*, were decreased (Fig. 5C).

**Figure 6:**
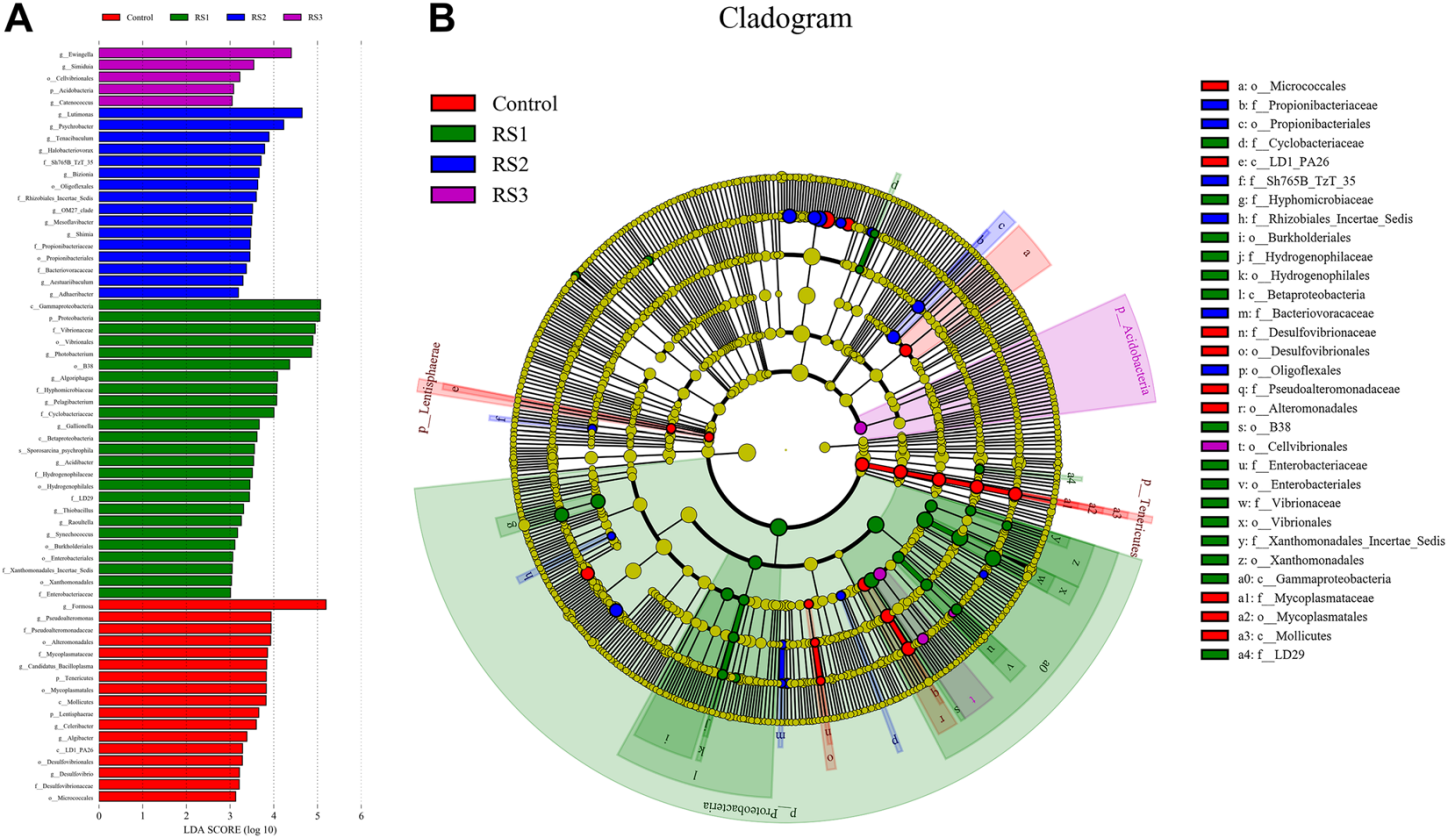
Inter-group variation of the intestine microbial of *L*. *vannamei* that were fed the control and three RS diets for 56 days. (**A**) LDA score of Lefse-PICRUSt. (**B**) Lefse cladogram.

The linear discriminant analysis (LDA) effect size (Lefse) was applied to detect the differential abundances of bacterial taxa among the four groups. The Lefse LDA scores showed that the abundances of 25, 16 and 5 taxa were increased in the RS1, RS2, and RS3 groups respectively, while that of 17 taxa were all decreased (Fig. 6A). There were 14 bacterial taxa that distinguished the four groups by LDA value, with 5, 3, 5, and 1 taxa in the control, RS1, RS2, and RS3 groups respectively (Fig. 6B). In detail, 2 classes, 4 orders, and 3 families were enriched in the control group, including Micrococcales (from the order level), Lentisphaerae (from the class level), Desulfobacterales (from the order to family level), Alteromonadales (from the order to family level), and Mollicutes (from the class to family level). One phylum, 2 classes, 6 orders, and 7 families were enriched in the RS1 group, including Proteobacteria (from the phylum to family level), Cyclobacteriaceae (from the family level), and LD29 (from the family level). Two orders and 4 families were enriched in the RS2 group, including Propionibacteriales (from the order to family level), Sh765B-TzT-29 (from the family level), Rhizobiales_Incertae_Sedis (from the family level), Bacteriovoracaceae (from the family level), and Oligoflexales (from the order level). One phylum and one order were enriched in the RS3 group, including Acidobacteria (from the phylum to order level).

### Changes in the intestine microbial metabolism

**Figure 7:**
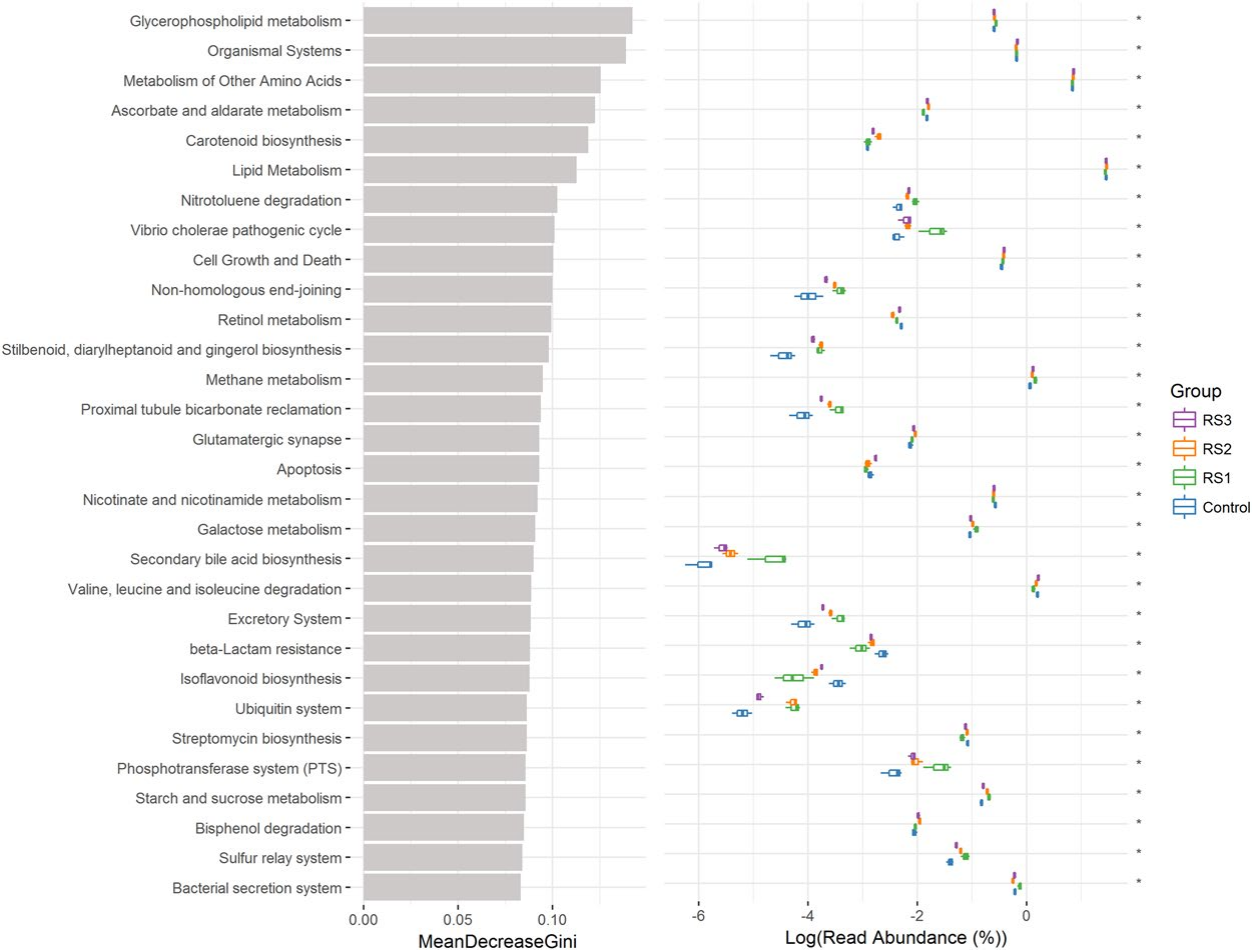
Intestine microbial metabolism of *L*. *vannamei* based on KEGG pathways analysis after the shrimp were fed the control and three RS diets for 56 days.

Based on random forest KEGG classification, the signalling of “nitrotoluene degradation”, “methane metabolism”, “stilbenoid, diarylheptanoid and gingerol biosynthesis”, “secondary bile acid biosynthesis”, and “starch and sucrose metabolism” were increased in the three RS groups, while that of “retinol metabolism”, “nicotinate and nicotinamide metabolism”, “isoflavonoid biosynthesis” and “beta-lactam resistance” were decreased (Fig. 7).

Shrimp fed the RS diet had a higher SCFAs content, including acetic acid (AA), propionic acid (PA), butyric acid (BA), and valeric acid (VA) than those of the control group (*P* < 0.05) (Table 2). The AA content was no difference among the three RS groups. The PA content was increased in the three RS groups with the increasing doses of RS. The BA content was highest in the RS3 group, while that in the RS1 and RS2 groups was not significantly different. The VA content was highest in the RS2 and RS3 groups, and they were statistically the same.

**Table 2:** The contents of SCFAs in the intestine of *L*. *vannamei* that were fed the control and three RS diets for 56 days (μg/g).

Items	Control	RS1	RS2	RS3
Acetic acid (AA)	69.79 ± 0.25^a^	74.07 ± 1.04^b^	75.15 ± 0.89^b^	74.88 ± 0.54^b^
Propionic acid (PA)	2.86 ± 0.14^a^	3.57 ± 0.23^b^	11.72 ± 0.57^c^	14.19 ± 0.41^d^
Butyric acid (BA)	1.59 ± 0.08^a^	3.45 ± 0.15^b^	3.27 ± 0.12^b^	8.18 ± 0.26^c^
Valeric acid (VA)	0.43 ± 0.04^a^	0.51 ± 0.06^b^	2.24 ± 0.11^c^	2.20 ± 0.08^c^

Values represent the mean ± SE (*n* = 3). The different letters (a–c) indicate significant differences (*P* < 0.05) among the groups.

### The intestine microbial were correlated with biochemical parameters

**Figure 8:**
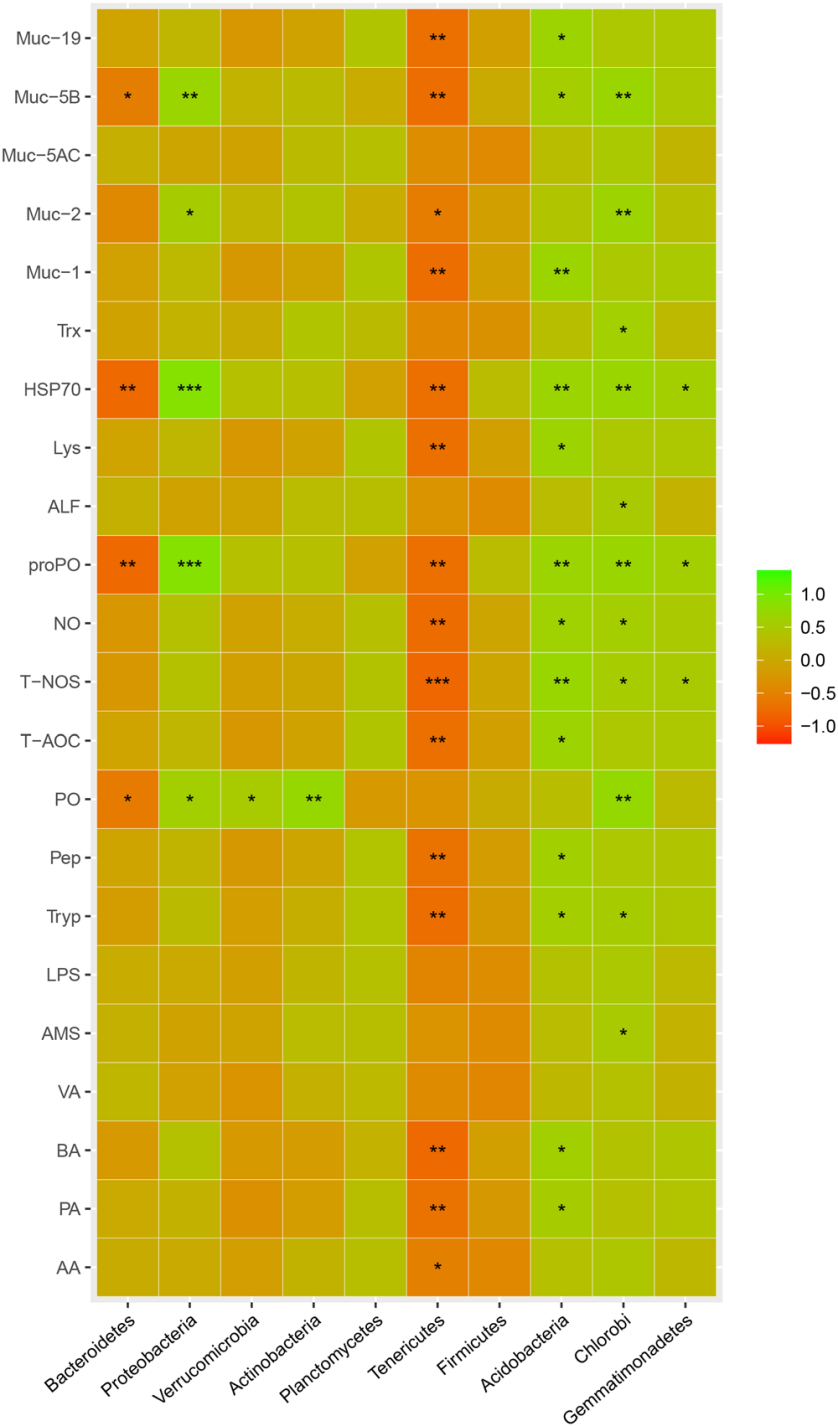
Significant correlation between intestine microbial phylum and shrimp health indices. Correlation coefficient is represented by different colours (green: positive correlation; red: negative correlation). *Data represent significantly negative or positive correlations (*P < 0.05, **P < 0.01, ***P < 0.001).

The correlations between the phylum abundance and the host biochemical parameters are shown in Fig. 8. The *proPO*, *HSP70*, *Muc-2*, and *Muc-5B* had positive correlations with the Proteobacteria. The PO had a positive correlation with the Proteobacteria, Verrucomicrobia, Actinobacteria, and Chlorobi. The Tyrp, T-NOS, NO, *proPO*, *HSP70*, and *Muc-5B* had positive correlations with the Acidobacteria and Chlorobi. The PA, BA, Pep, T-AOC, *Lys*, *Muc-1*, and *Muc-19* had positive correlations with the Acidobacteria. The AMS, *ALF*, *Trx*, and *Muc-2* had positive correlations with the Chlorobi. The T-NOS, *proPO*, and *HSP70* had positive correlations with the Gemmatimonadetes.

## Discussion

The intestine health influences the shrimp health because intestine mucosa provides an important barrier to pathogens. Intestine health is related to its structural integrity, mucus components and immune molecules^2^. The intestine mucosa structure of shrimp can be damaged by environmental stress and pathogen challenges^16^, which may increase the risk of pathogen invasion and impair the host immunity^17^. The nutrient uptake of the intestine mainly relies on its epithelium and microvilli. Here, we examined the effects of dietary RS on intestine mucosa morphology and the digestive enzymes activity of the shrimp. We found that shrimp fed the RS diet had a higher epithelium height and digestive enzymes activity. Hence, dietary RS improved the intestine histological structure and promoted its digestive capacity, which is likely to benefit nutrient absorption.

Antibacterial molecules play vital roles in the immune homeostasis of shrimp. Mucus is located on the surface of the intestine mucosa, which can lubricate the intestine tract and resist pathogenic infection^18^. Muc is the main antimicrobial component of intestine mucus^19,20^. It was reported that dietary RS could increase the *Muc-4*, *Muc-5AC*, and *Muc-12* genes in the intestine of pigs^21^. As important immune parameters, *ALF*, *proPO*, *Lys*, NOS and NO function as strong antibacterial forces against pathogenic microbes^22–25^. In this study, the PO and T-NOS activity, NO content, and the expression levels of the *propO*, *ALF*, *Lys*, *Muc-1*, *Muc-2*, *Muc-5AC*, *Muc-5B* and *Muc-19* genes were increased in the three RS groups, which suggested that dietary RS could improve the intestine mucin and antibacterial capacity of the host towards resisting pathogenic infection.

The environment induces oxidative stress in aquatic animals^26^. Organisms have evolved an antioxidant defence system to counteract oxidative stress, including antioxidant enzymes and proteins. The total status of antioxidant enzymes can be reflected by T-AOC activity^27^. As important antioxidant proteins, HSPs can increase endogenous peroxidase activity to eliminate reactive oxygen species (ROS)^28^; Trx acts as a peroxiredoxin substrate that functions in cell redox homeostasis^29^. In this study, dietary RS increased the T-AOC activity and the *HSP70* and *Trx* gene expression in the shrimp intestine. These phenomena revealed that dietary RS could enhance the intestine antioxidant function, which might contribute to the regulation of the redox status of the intestine.

Shrimp microbial are important for its host health, which can be regulated by diet^30–32^. Here, we found that dietary RS improved the composition of intestine microbial of the shrimp and increased the microbial carbon source and lipid metabolism, including starch, sucrose, SCFAs, bile acid, and gingerol. SCFAs can create an acidic environment in the intestine of shrimp, which accelerate increase of beneficial bacteria and reduce the proliferation o pathogenic bacteria^9^. Thus, dietary RS can increase the diversity and beneficial metabolites of intestine microbial to improve the intestine health of shrimp. After the shrimp fed the RS diets for 56 days, the bacterial phylum Proteobacteria abundance increased, while the Bacteroidetes abundance decreased; these two phylum are the dominant intestine bacterial of the shrimp during its growth stages^16,33–35^. The abundance of Actinobacteria was enriched in the three RS groups. The correlation analysis of the bacterial phylum and its host health indicators showed that Proteobacteria had a positive correlation with immune indices (PO, *proPO*, *HSP70*, *Muc-2*, and *Muc-5B*). Additionally, the PO had a positive correlation with the Actinobacteria abundance. Actinobacteria are good elaborators of pharmaceutical products, including antibiotics and antimicrobial agents^36^. Hence, forecasts of these phylum bacterial contributed to the intestine health of the host, particularly in antibacterial, antioxidant, and mucin secretion.

Some beneficial bacterial genera were enriched after the shrimp fed with the RS diets, including *Lutimonas*, *Ruegeria*, *Shimia*, *Mesoflavibacter*, and *Mameliella*. *Lutimonas* is a strictly aerobic heterotrophic nitrifying bacterium for the degradation of ammonia^37^. *Ruegeria* and *Shimia* are both members of the Roseobacter clade, with essential metabolic capabilities, and are involved in protein utilization^38^. *Ruegeria* also have high phosphodiesterase and phosphomonoesterase activities that contribute to the degradation of phosphate triester compounds in aquatic environments^39,40^. *Mameliella* play important roles in the degradation of aromatic compounds^41,42^ and can accumulate beneficial poly-β-hydroxybutyrate (PHB) granules in cells^43^. *Mesoflavibacter* can produce zeaxanthin that have strong antioxidant and anticancer properties^44,45^. Interestingly, based on the KEGG pathway analysis, “nitrotoluene degradation” and “methane metabolism” were increased in the three SA groups, which indicated that dietary RS contribute to the enrichment of the beneficial bacteria that are involved in degrading toxins and producing beneficial metabolites. Furthermore, opportunistic pathogens, such as *Formosa* and *Pseudoalteromonas*, were absent in response to dietary RS. *Formosa* was enriched in the intestine of *L*. *vannamei* when the shrimp were subjected to ammonia and nitrite stress, respectively^33^. *Pseudoalteromonas* can cause high mortality in *Portunus pelagicus*^46^. Therefore, this study illuminated that dietary RS could optimize the intestine microbial composition of *L*. *vannamei* and decrease the risk of pathogen invasion to the host.

In conclusion, dietary RS improved the intestine health of *L*. *vannamei*. Of these, dietary RS improved the intestine mucosa morphology, enhanced the intestine digestive and immune capacity of the shrimp, including mucus, antibacterial, and antioxidant enzyme activity and/or gene expression. Furthermore, dietary RS also regulated the composition and SCFAs contents of the intestine microbial, and there was a positive correlation between some bacterial phylum and the host health indices.

## Materials and Methods

### Ethics statements

The collection and handling of the animals in this study was approved by the Animal Care and Use Committee at the Chinese Academy of Fishery Sciences (CAFS), and all experimental animal protocols were carried out in accordance with national and institutional guidelines for the care and use of laboratory animals at the CAFS (No. 2016TS07).

### Diet preparation

The source of RS in this study was high amylose maize (HI-MAIZE^®^ 260), which was purchased from National Starch Industrial (Shanghai) co., LTD. According to the supplier, this starch was obtained directly from high amylose maize seeds, the RS contents was 80%, and it could be classified as an RS2 type; no other exogenous ingredients were added. Four experimental diets were prepared and differed in RS content: 0 g/kg (Control), 10 g/kg (RS1), 30 g/kg (RS2) and 50 g/kg (RS3), and the feed formula is given in Table S1. In the feed ingredients of the three RS groups, we used RS instead of wheat flour. The diet preparation was referenced to Duan *et al*.^47^ Moisture, crude protein, crude lipid and ash of the experimental diets were determined using a standard method of Association of Official Analytical Chemists (AOAC) (1995).

### Shrimp and rearing conditions

Healthy juvenile *L*. *vannamei* were randomly selected from a local farming pond in Shenzhen, China; the average weight was 3.2 ± 0.3 g. The shrimp were temporarily reared and acclimated for 7 days before the feeding trial experiment. The rearing water of the shrimp was sand-filtered seawater, which was aerated with two stones. The parameters of the water quality were pH 8.4, salinity 30, temperature 30 ± 0.5 °C, and dissolved oxygen 6.0 ± 0.5 mg l^–1^. The shrimp were fed daily at 5% of body weight. Change one third of the water each day.

After acclimation, the shrimp were divided into four groups: Control, RS1, RS2, and RS3, and each group was fed with the corresponding experimental feed. Each group contained three 500 L replicate tanks, and 40 shrimp per tank. The culture condition was in accord with the acclimation stage. All tanks were fed three times per day at 7:00, 12:00 and 18:00 for 56 days. Uneaten feed and faeces were cleared out from the tanks. At 56 days, the intestine of the shrimp of each tank were sampled individually and were snap-frozen in liquid nitrogen for biochemical, gene expression, and microbiome analysis.

### Histological analysis

The intestines from three shrimp per tank were extracted at 56 days and were stored in Davidson solution for 24 h. After being rinsed with running water for 8 h, and the embedded lumps were put into 70% ethanol overnight. Then, the samples were dehydrated in a series of ethanol solutions (80%, 90%, and 100%), followed by acetone and xylene transparent and paraffin embedding. The tissue was sectioned in a microtome (Leica, RM2016, Wetzlar, Germany) to a 4 μm thickness. After staining with haematoxylin and eosin (HE), stained sections were observed and photographed under light microscope (Olympus, Tokyo, Japan). The intestine EH and WT were detected six sites randomly.

### Biochemical analysis

The intestine from three shrimp per tank were extracted at 56 days and homogenized to 10% ratio with 0.9% saline solution, then centrifuged (3500 rpm, 4 °C, 10 min). The supernatant was immediately detected for biochemical parameters using a microplate reader (Bio-Rad, USA). AMS, Lip, Tryp, Pep, PO, T-AOC, T-NOS, and NO were measured using related commercial assay kits (Nanjing Jiancheng Institute, China) according to the manufacturer’s protocols. The total protein concentration in tissue homogenates was measured using a Coomassie brilliant-blue protein assay kit (Jiancheng, Nanjing, China). The assays were all run in three replicate samples.

### Gene expression analysis

The total RNA from the intestine of three shrimp per tank was extracted at 56 days using TRIzol Reagent (Invitrogen, USA); then, the genome DNA was eliminated using RQ1 RNase-Free DNase (Promega, USA). The total RNA (8 μg) was reverse transcribed to cDNA using PrimeScript™ RT Reagent Kit (Takara, China) according to the manufacturer’s protocol. Real-time RT-qPCR was conducted in a LightCycler480 System using a SYBR^®^ Premix Ex Taq™ II Kit (TaKaRa, Japan). *β-actin* gene of *L*. *vannamei* was chosen as an internal control, and the specific primer sequences of the target genes were designed using Primer Premier 5.0 software and are listed in Table S2. RT-qPCR was carried out using the method of Duan *et al*.^47^. The relative gene expression was calculated by the 2^*−ΔΔCT*^ comparative *C*_*T*_ method and is shown as the fold-change in comparison to the control group.

### Intestine microbiome analysis

The intestine microbial DNA of six shrimp per tank was extracted at 56 days using a PowerSoil™ DNA Isolation Kit (Mo Bio Laboratories, Inc., Carlsbad, CA) according to the manufacturer’s protocol, and analyzed using 1.0% agarose gel electrophoresis. DNA concentration and quality were checked using a NanoDrop Spectrophotometer. The V4 region of the bacterial 16S rRNA gene was amplified using the barcoded fusion primers 515F and 806R (Table S2). The PCRs were each 20 μL, containing the FastPfu polymerase 0.4 μL, dNTPs (2.5 mM) 2 μL, 5 × FastPfu buffer 4 μL, each primer (5 μM) 0.8 μL, and template DNA 10 ng. The PCR programs were 1 cycle of 95 °C for 5 min, 27 cycles of 95 °C for 30 s, 55 °C for 30 s, and 72 °C for 45 s, followed by 72 °C for 10 min. The PCR products were purified by a PCR purification kit (Qiagen), then were sequenced with an Illumina HiSeq platform. The sequences analysis was referenced to Duan *et al*.^33^.

### Intestine SCFAs content analysis

SCFAs content, including AA, PA, BA, and VA from the intestine of three shrimp per tank were detected at 56 days using gas chromatography, which was referenced to Weitkunat *et al*.^48^.

### Statistical analysis

The data were presented as the mean ± SE, and statistically analyzed using one-way ANOVA, followed by Duncan’s multiple range testing (SPSS v22.0). Correlation between the host intestine health indices and the bacterial relative abundance by phylum (top ten) was estimated using the Pearson test (−0.8 < *R* < 0.8). *P* < 0.05 was regarded as statistically significant.

## Supplementary information

Supplementary information
