# Supplementary material for: Changes in the intestine microbial, digestion and immunity of Litopenaeus vannamei in response to dietary resistant starch
Source: Sci Rep. 2019 Apr 23;9:6464. doi: 10.1038/s41598-019-42939-8 (PMC6478684; doi:10.1038/s41598-019-42939-8)
Supplement: Supplementary file 1 — Supplementary information [file 41598_2019_42939_MOESM1_ESM.doc]

**Changes in the intestine microbial, digestion and immunity of *Litopenaeus vannamei* in response to dietary resistant starch**

**Yafei Duan, Yun Wang, Qingsong Liu, Dalin Xiong, Hua Li, Hongbiao Dong, Jiasong Zhang***

Key Laboratory of South China Sea Fishery Resources Exploitation & Utilization, Ministry of Agriculture and Rural Affairs; Key Laboratory of Fishery Ecology and Environment, Guangdong Province; South China Sea Fisheries Research Institute, Chinese Academy of Fishery Sciences, Guangzhou 510300, PR China.

Correspondence and requests for materials should be addressed to J.S. Z. (jiasongzhang@hotmail.com)

**Table S1.** Formula ingredients and nutrient values of the experimental diets.

| Group | Control | RS1 | RS2 | RS3 |
| --- | --- | --- | --- | --- |
| Ingredients (g/kg dry matter) | | | | |
| White fish meal^a^ | 250.0 | 250.0 | 250.0 | 250.0 |
| Soybean meal^b^ | 180.0 | 180.0 | 180.0 | 180.0 |
| Peanut bran^b^ | 164.0 | 164.0 | 164.0 | 164.0 |
| Wheat flour^b^ | 230.0 | 220.0 | 200.0 | 180.0 |
| Beer yeast^b^ | 50.0 | 50.0 | 50.0 | 50.0 |
| Krill meal^b^ | 50.0 | 50.0 | 50.0 | 50.0 |
| Soybean lecithin^c^ | 10.0 | 10.0 | 10.0 | 10.0 |
| Fish oil^d^ | 10.0 | 10.0 | 10.0 | 10.0 |
| Soybean oil^b^ | 10.0 | 10.0 | 10.0 | 10.0 |
| Choline chloride (50%) | 5.0 | 5.0 | 5.0 | 5.0 |
| Ca(H_2_PO_4_)_2_ | 10.0 | 10.0 | 10.0 | 10.0 |
| V_C_-phosphate ester^e^ | 1.0 | 1.0 | 1.0 | 1.0 |
| Vitamin premix^f^ | 10.0 | 10.0 | 10.0 | 10.0 |
| Mineral premix^g^ | 10.0 | 10.0 | 10.0 | 10.0 |
| Resistant starch (RS)^h^ | 0 | 10.0 | 30.0 | 50.0 |
| Sodium alginate | 10.0 | 10.0 | 10.0 | 10.0 |
| Total | 1000 | 1000 | 1000 | 1000 |
| Nutrient value (g/kg dry weight)^i^ |  |  |  |  |
| Crude protein | 398.4 | 408.1 | 398.2 | 403.4 |
| Lipid | 71.5 | 72.1 | 71.7 | 71.9 |
| Ash | 119.5 | 122.1 | 121.3 | 117.2 |
| Moisture | 105.6 | 115.3 | 105.8 | 109.1 |

^a^ Imported from N.E.L.T.O. Australia Pty Ltd.

^b^ Zhuhai Shihai Feed Corporation Ltd., Zhuhai, China.

^c^ Kemin Industries (Zhuhai) Ltd., Zhuhai, China.

^d^ Imported from New Zealand (Bakels Edible Oils Ltd, Mt Macnganui).

^e^ Guangzhou Chengyi Company Ltd., Guangzhou, China.

^f^ Vitamin premix (g kg^-1^): retinyl acetate, 2.5; cholecalciferol, 6.25; all-rac-atocopheryl acetate, 75; menadione, 2.5; thiamin, 0.25; riboflavin, 1; D-calcium pantothenate, 5; pyridoxine HCl, 0.75; cyanocobalamin, 2.5; niacin, 2.5; folic acid 0.25; biotin 2.5; meso-inositol, 379; cellulose, 500.

^g^ Mineral premix (g kg^-1^): KCl, 90; KI, 0.04; NaCl, 40; CuSO_4_-5H_2_O, 3; ZnSO_4_-7H_2_O, 4; CoSO_4_-7H_2_O, 0.02; FeSO_4_-7H_2_O, 20; MnSO_4_-H_2_O, 3; MgSO_4_-7H_2_O, 124; CaHPO_4_-2H_2_O, 500; CaCO_3_, 215.

^h^ Resistant starch (RS) was purchased from National Starch Industrial (Shanghai) co., LTD..

^i^ Measured values.

**Table S2.** Primer sequence used in this study.

| Primer name | Sequence (5'-3') | GenBank accession number |
| --- | --- | --- |
| 515F | GTGYCAGCMGCCGCGGTAA | Parada et al., 2015 |
| 806R | GGACTACNVGGGTWTCTAAT | Apprill et al., 2015 |
| *proPO*-F | CAATGACCAGCAGCGTCTTC | AY723296 |
| *proPO*-R | CACGGAAGGAGGCGTATCAT |
| *ALF*-F | GGTGTTCCTGGTGGCACTCT | GQ227486 |
| *ALF*-R | AGCTCCGTCTCCTCGTTCCT |
| *Lys*-F | GTTCCGATCTGATGTCCGATG | AY170126 |
| *Lys*-R | AAGCCACCCAGGCAGAATAG |
| *HSP70*-F | CAACGATTCTCAGCGTCAGG | AY645906 |
| *HSP70*-R | ACCTTCTTGTCGAGGCCGTA |
| *Trx*-F | TTCCTGAAGGTGGATGTGGA | EU499301 |
| *Trx*-R | AGTTGGCACCAGACAAGCTG |
| *Muc-1*-F | GGCTCGGAAGTTGGCGATGATG | Duan et al., 2018 |
| *Muc-1*-R | CGATGGCTCAATGGCGAAGAGG |
| *Muc-2*-F | TGCCAGCCACGTCCTCCTTG | Duan et al., 2018 |
| *Muc-2*-R | CCGCAGCCGAGGCAGTCC |
| *Muc-5AC*-F | AGCAGGACTTCAACGACTACAACAG | Duan et al., 2018 |
| *Muc-5AC*-R | GCGCGACGCCGATGATGG |
| *Muc-5B*-F | CTTGACGCATACGCTCAGGTTCC | Duan et al., 2018 |
| *Muc-5B*-R | TCCGCCGCCTTCATCCTCTG |
| *Muc-19*-F | GAAGAGGAGGAAGAGGACGAGGAG | Duan et al., 2018 |
| *Muc-19*-R | GGACCACCAGGCACAAGAACATC |
| *β-actin*-F | GCCCTGTTCCAGCCCTCATT | AF300705 |
| *β-actin*-R | ACGGATGTCCACGTCGCACT |

*proPO*, prophenoloxidase; *Lys*, lysozyme; *HSP70*, heat shock protein 70; *Trx*, thioredoxin 1; *Muc-1*, mucin-1; *Muc-2*, mucin-2; *Muc-5AC*, mucin-5AC; *Muc-5B*, mucin-5B; *Muc-19*, mucin-19.

Parada, A. E., *et al*. Every base matters: assessing small subunit rRNA primers for marine microbiomes with mock communities, time series and global field samples. *Environ. Microbiol.* **18**, 1403-1414 (2016).

Apprill, A., *et al*. Minor revision to V4 region SSU rRNA 806R gene primer greatly increases detection of SAR11 bacterioplankton. *Aquat. Microb. Ecol.* **75**, 129-137 (2015).

Duan, Y. F., Liu, Q. S., Zhang, J. S., Wang, Y. & Xiong, D. L. Impairment of the intestine barrier function in *Litopenaeus vannamei* exposed to ammonia and nitrite stress. *Fish Shellfish Immunol.* **78**, 279-288 (2018).
